# Withaferin-A attenuates diabetes mellitus induced male reproductive dysfunction mediated by ERα in brain and testes of Swiss albino mice

**DOI:** 10.1038/s41598-023-44904-y

**Published:** 2023-10-17

**Authors:** Kalpana Baghel, Zaffar Azam, Rashmi Srivastava, Neelima Gupta, Naveen Kango

**Affiliations:** 1 Department of Microbiology, School of Biological Sciences, Dr. Harisingh Gour Vishwavidyalaya (A Central University), Sagar, MP 470003 India; 2 Department of Zoology, School of Biological Sciences, Dr. Harisingh Gour Vishwavidyalaya (A Central University), Sagar, MP 470003 India; 3 Department of Zoology, University of Allahabad, Prayagraj, UP 211002 India; 4 Dr. Harisingh Gour Vishwavidyalaya (A Central University), Sagar, MP 470003 India

**Keywords:** Biochemistry, Physiology, Systems biology, Zoology, Diseases, Endocrinology, Medical research

## Abstract

Diabetes mellitus (DM) is a chronic metabolic disease, characterized by persistent hyperglycemia resulting from diminished insulin secretion or insulin resistance. The present study evaluated the ameliorative effects of Withaferin-A (WA) on DM-induced reproductive dysfunction in mice. For the same, mice were intraperitoneally injected with Streptozotocin (STZ), (40 mg/kg/day) for 5 consecutive days to induce DM. Mice were then treated with WA (8 mg/kg/day) in normal and diabetic conditions (STZ + WA). Next, blood glucose levels, oral glucose tolerance, intraperitoneal insulin tolerance, oxidative stress and reproductive parameters were estimated. For reproductive performance, immunofluorescent localization of gonadotropin-releasing hormone (GnRH-I) and estrogen receptor alpha (ERα) in the preoptic area and paraventricular nucleus region of hypothalamus and ERα in testes was performed. STZ-induced diabetes triggered reproductive dysfunctions as mediated by low GnRH-I and ERα in the brain and ERα in the testes along with declined testosterone and estradiol levels. Treatment with WA significantly reduced the blood glucose levels and enhanced glucose clearance accompanied by reduced oxidative stress in the brain, pancreas and testes as indicated by the low levels of H_2_O_2_ and MDA in diabetic mice treated with WA (STZ + WA). This study reports, for the first time, that WA can efficiently ameliorate DM-induced reproductive dysfunctions by enhancing endogenous testosterone, estrogen and increased GnRH-I and ERα in the brain and ERα in the testes of DM-induced male mice.

## Introduction

Diabetes Mellitus (DM) is a metabolic disorder determined by persistent hyperglycemia. DM-induced hyperglycemia is characterized by low insulin release, declined glucose use, and high glucose production^1^. Prevalence of DM is growing rapidly as it affects more than 415 million adults globally. WHO reported that in 2019, 1.5 million deaths were directly related to diabetes, and 48% of those deaths happened before the age of 70^2,3^. Moreover, the incidence of DM in males is greater than in females. Hyperglycemic state due to pancreatic β-cells degradation causes many complications including reproductive dysfunction^4^, along with declining the hypothalamic neuronal number^5^. A study reported that over 50% of diabetic males suffer from erectile dysfunction (ED)^6^. The number of ED cases worldwide is anticipated to reach 322 million by the year 2025^7,8^. Patients with prolonged duration of DM and upper age are more susceptible to sexual and reproductive impairment or dysfunctions which are induced by impaired spermatogenesis, reduced synthesis and secretion of testosterone and altered glucose metabolism in Sertoli cells^9,10^. Reproductive activities are regulated by sex steroid hormones such as testosterone and estrogen in males and females. Males tend to have lower estrogen levels than females but it plays a critical role in male reproductive health, particularly in the regulation of spermatogenesis and spermiogenesis. Estrogen signalling is mediated by estrogen receptor alpha (ERα) and estrogen receptor beta (ERβ). The significance of estrogen in the male reproductive system is proven as mice lacking functional estrogen receptors or aromatase gene (cyp 19 gene) become infertile. Predominantly, estrogen receptor knock-out (ESR-KO) mice became sterile due to the defectiveness in growth and functions of efferent ductules^11^. In the mammalian system, it has been reported that an estrogen-responsive mechanism is essential for the regulation of the male reproductive system since the inactivation of ERα and ERβ leads to reproductive dysfunctions^12,13^. It has been shown that ERα is involved in the regulation of reproductive activity. The distribution of both receptors in the brain was typically similar while nuclear immunoreactivity (*ir*) of ERα was predominant in the hippocampus, preoptic area, and the majority of the hypothalamus^14–16^. Both ERs regulate the differentiation, growth of axons, dendrites in the brain, and reproductive behaviour of organisms including mammals. The primary reproductive organs of males are the testes, where the estrogen gets synthesized in Leydig cells from androgens in the presence of an aromatase enzyme. Localization of ERs has been demonstrated in the male genital system of various species, including primates (humans and non-humans)^17,18^, rodents (mice and rats)^19,20^, domestic animals (cats, dogs, porcine, goat, and sheep)^21,22^.

Oxidative stress has harmful impact on health and induces many disorders including diabetes, cancer, infertility, and neurodegenerative diseases^23,24^. There are many bioactive compounds sourced from medicinal plants which are known to overcome oxidative stress due to their scavenging and notable antioxidant activities. Several conventional herbs are easily accessible, regarded as safe, and highly beneficial for the prevention of many illnesses. One such traditional medicinal plant *Withania somnifera* (WS), also known as Ashwagandha (Indian Ginseng), a member of the Solanaceae family has been used for many years to treat various health complications e.g., rheumatoid arthritis, immunological problems, cardiovascular disease, and infertility^25^. WS encompasses several bioactive compounds such as withanolide-D, flavonoids, alkaloids, and steroidal lactone like Withaferin-A (WA). WA, a bioactive compound of Ashwagandha, is a primary bioactive metabolite and is also reported to relieve pancreatitis, an exocrine disorder^26^.

However, there is hardly any conclusive study that reports the pharmacological effect of WA in DM-triggered reproductive dysfunction mediated by ERα. Thus, the current study for the first time aimed to investigate the deleterious effects of diabetes and its amelioration by WA administration in DM-induced reproductive dysfunction mediated by ERα and oxidative stress in male mice.

## Materials and methods

### Animals and treatments

At least two weeks prior to the initiation of the experiment, 8 weeks old, Swiss albino male mice (weight 25–30 g), n = 4 mice/cage were acclimatized under specific pathogen-free (SPF) conditions with 12 h light/dark cycle, 40–60% humidity and 24 °C temperature. Mice were provided with ad libitum access to autoclaved drinking water and a standard laboratory chow diet. Mice were then arbitrarily divided into four groups (n = 10 in each group). Group-1 was kept as control (C) in which 1% DMSO + 99% PBS was administered as a vehicle intraperitoneally (i.p.). Group-2 received streptozotocin (40 mg/kg/day) for five consecutive days i.p.), and was indicated as STZ. Group-3 was treated with WA alone (8 mg/kg/day i.p.) for 60 days (WA prepared in 1% DMSO + 99% PBS), while Group-4 indicated as (STZ + WA) was induced diabetes with STZ and treated with WA (8 mg/kg/day) for 60 days. Day 1 was defined as the initial injection of STZ and the animals were sacrificed on day 64. The dose of Withaferin-A (HPLC grade, > 98% purity) procured from Aptus Therapeutics, Hyderabad, India, was chosen on the basis of literature describing its therapeutic properties and safety profile^27–29^. At the end of the experiment, mice were euthanized by carbon dioxide asphyxiation and desired tissues were harvested for further analysis. The experiment was repeated twice for the validation of the results. The animals were procured from the College of Veterinary Science and Animal Husbandry, Mhow, (M.P.), India. The experiments were conducted in accordance with the principles, recommendations, and protocols approved by the Institutional Animal Ethics Committee (IAEC), Doctor Harisingh Gour Vishwavidyalaya, Sagar, Madhya Pradesh, India (Vide No. 379/CPCSEA/IAEC-2021/030). Authors have complied with the ARRIVE guidelines and all possible efforts were made to lessen the animal suffering.

### Development of STZ-induced DM mice model

Diabetes was induced with a high fat diet for two weeks followed by injecting multiple low doses of STZ, (Sigma, St. Louis, MO), 40 mg/kg/day for 5 consecutive days^30,31^. Prior to STZ injection, mice were kept for 12 h fasting. STZ was prepared fresh by dissolving in 0.01 M cold sodium citrate buffer (pH 4.5) prepared immediately before use. Doses were injected according to the body weight of the mice. Fasting glucose was measured per week till the completion of the experiment. Mice with blood glucose levels > 11.1 mmol/L were considered as diabetic for subsequent experiments. In addition to the regular monitoring of the body weight and blood glucose level, the health condition of the mice was evaluated on daily basis according to the body condition scoring including the classic changes in physical appearance i.e., bulging or sunken eyes, nasal or ocular discharge, rough coat, lethargic condition and hunched back^32,33^.

### Blood collection and tissue harvesting

The body weight of mice was recorded before anaesthetising with ketamine (100–150 mg/kg) and xylazine (5–16 mg/kg) for collecting blood through retro-orbital sinus^34^. For serum isolation, the blood was allowed to clot in tubes by keeping it undisturbed at room temperature (RT) for 20 min. The serum was then separated by centrifuging the blood in a refrigerated centrifuge at 700×*g* for 25 min and stored at − 20 °C for further hormonal analysis. Four mice from each group were euthanized by CO_2_ asphyxiation and the brain, pancreas, and testes were excised from the body and instantly frozen at − 80 °C for the measurement of oxidative stress and antioxidant enzyme activities. The remaining six mice from each group were anaesthetized and perfused with 0.02 M phosphate buffer saline (PBS) followed by Zamboni’s fixative through the left ventricle of the heart. The brain, pancreas, and testes were harvested for further histopathological and immunohistochemical analysis.

### Measurement of fasting blood glucose levels

Blood glucose was estimated routinely (per week) for the assessment of diabetes^35^. Blood glucose level of overnight-fasted mice was measured by nicking the end of the tail. A small amount of blood was taken from the tail vein and put into a glucometer to measure the amount of glucose (mg/dL).

### Oral glucose tolerance test (OGTT)

Next, we performed OGTT to evaluate glucose tolerance and physiological functions of the pancreas along with a comparison of the active clearance of glucose burden between WA-treated and diabetic mice. After overnight fasting, 2 g/kg of glucose was administered orally to the mice for the assessment of their glucose tolerance. After the administration of glucose, blood glucose concentrations were checked at 0, 30, 60, and 90 min using a mechanized counter of the glucometer with digital readings. The readings were converted to mmol/L using the formula: mmol/L (glucose) = (mg/dL value)/18. The area under curve (AUC) for estimating blood glucose levels and OGTT was determined using the standard trapezoid rule^29^.

### Intraperitoneal insulin tolerance test (IPTT)

Insulin (0.75 U/kg, i.p.) was injected and blood glucose levels were observed at 15, 30, 60, 90, and 120 min after injection and the blood glucose concentrations were assessed after 4 h fasting with an Accu-check glucometer (Roche Diagnostics, Germany). Further, the insulin resistance index (HOMA-IR) was calculated using fasting serum insulin and glucose levels (available from www.dtu.ox.ac.uk/ homacalculator).

### Histology of pancreas, brain, and testes

Post-completion of the experiment, mice were sacrificed and brain, pancreas, and testes were excised, weighed, and kept in Zamboni’s fixative for 24–48 h. All the tissues were dehydrated in ascending grades of alcohol series, cleared in xylene and embedded in paraffin wax. Brain, pancreas, and testes sections were cut using a rotary microtome (Leica RM 2125RTS, Wetzlar, Germany) by keeping the thickness at 8 μm, 5 μm and 6 μm, respectively. Cresyl violet (CV) staining was applied to the brain sections to observe and count the number of neurons in POA and PVN of the hypothalamus. Hematoxylin and Eosin (H&E) staining was performed for histological observations of the pancreas and testes. Histopathological alterations in the pancreas were observed by 6 randomly selected sections from respective groups. Insulitis was measured according to the percentage of islets in each category of infiltration in accordance with a previously defined method^30^. Six histological sections were randomly selected from each group and were used for measurement of the diameter of seminiferous tubules by using an occulometer and micrometer (ERMA, Japan). The testicular volume was also calculated using Bissonett’s formula: 4/3πab^2^ (where, a = half of the long axis and b = half of the short axis) as defined earlier^36,37^. Testicular density was measured by the following formula: testes weight/volume and expressed as (g/cm^−3^)^38^. The sexual maturity of mice was calculated by the formula of gonado-somatic-index (GSI): GSI = (weight of testes/body weight) × 100^39^.

#### Johnsen score

Johnsen score, used for the evaluation of the testicular cells, was performed for quantifying spermatogenesis using the 10-point scoring system in the seminiferous tubules indicating different spermatogenesis stages as described earlier by Johnsen, 1970^40^. Randomly, six sections from each group were selected for measuring Johnsen score under 400X magnification.

#### Measurement of the seminiferous tubule basement membrane thickness

The testes sections were enlarged under high magnification (400×) using a compound microscope. Randomly, 10 seminiferous tubules were selected from each section for the estimation of thickness of the basement membrane around the seminiferous tubules^41^.

#### Measurement of the seminiferous tubule epithelial height

The epithelium height was measured in 10 seminiferous tubules located in central and peripheral regions from each section that were either round or nearly round at 400X using an occulometer. The thickness of the seminiferous tubule’s epithelium was measured from the spermatogonium near the basement membrane to the spermatids^41^.

## Sperm counting

Mice spermatozoa were collected from the cauda epididymis. The epididymis was minced in 2 ml of PBS (pH 7.2) for making sperm suspension. The suspension was then filtered through 80 µm nylon mesh to eliminate tissue trashes. An aliquot of 500 µl from the stock sperm suspension (1 ml) was diluted (1:20) by adding PBS (pH 7.2). Next, sperm count and motility in sperm suspensions were estimated. These suspensions were directly positioned on the pre-warmed slides and were examined at 400×. Motile sperms that move easily in a forward direction were considered while those vibrating or rotating in the same location were not included in this category. After swim-out, the remaining sperm suspensions were diluted in water to retain movement for sperm count. Subsequently, using a Neubauer Chamber slide and a light microscope (400×), the number of sperm heads were counted as per the standard method. Sperm viability was assessed by dye exclusion method using 0.5% w/v Eosin yellow (Sigma Aldrich), and the proportion of spermatozoa that did not integrate the dye (i.e., viable spermatozoa) was estimated^42^.

### Immuno-fluorescent localization of GnRH-I, ERα, and Caspase-3

To study the immunostaining of GnRH-I, ERα, and Caspase-3, 10 and 5 µm thick sections of brain (GnRH-I) and testes (ERα and Caspase-3) were cut, respectively, mounted on glass slides and dried overnight at 37 °C. Sections were then incubated in 10 mM antigen retrieval buffer (pH 6.0) for 6–8 min, washed with triton X-100 for 12 min, rinsed in PBS and finally flooded with 5% normal goat serum (NGS) for 60 min. The sections were then incubated with primary polyclonal antibodies for binding with ERα (Abcam-3575, Cambridge, UK) at a dilution of 1:200 overnight in a humidified chamber at 4 °C. For detecting the GnRH-I receptor and Caspase-3, monoclonal primary antibodies sc-32292 and sc-7272 (Santa Cruz Biotechnology, CA, USA) with dilutions 1:200 and 1:300 were used, respectively. Next day, slides were incubated with the secondary antibody fluorescein iso-thiocyanate (conjugated goat anti-mouse/rabbit immunoglobulin) at a dilution of 1:200 for 90 min at 4 °C. The sections were counterstained with 4′, 6-diamidino-2-phenylindole (DAPI), (Sigma Aldrich, St. Louis, USA) for 6 min at 37 °C, mounted in glycerine-based mounting media and visualized with a fluorescent microscope (EVOSM 5000, Thermo Fisher). Negative control slides were processed without adding primary antibodies^37^.

#### Immuno-histochemistry of p53

IHC for p53 in testes sections (5 μm) was done using a Vectastain ABC kit (Burlingame, CA 94010, USA). For immuno-localization of p53, testes sections were incubated with a mouse monoclonal antibody (sc-126, Santa Cruz Biotechnology, CA, USA) against p53 (1:100) for 36 h as described earlier^43^. After incubation, slides were washed with PBS and then incubated with biotinylated goat anti-rabbit IgG (1:50) for 90 min. Then slides were rinsed in PBS and stained with 3, 3’-diaminobenzidine tetrachloride solution (1% w/v) containing 0.3% H_2_O_2_ and 1% nickel chloride for detecting the bound antibodies. Further, slides were dehydrated in graded ethanol series and air-dried for observation. The negative control was run without adding primary antibodies and no positive immunoreactivity was detected on these slides.

#### Semi-quantitative analysis of GnRH-I, ERα, Caspase-3 and p53

The semi-quantitative analysis of GnRH-I, ERα, Caspase-3 and p53 in the immuno-positive cells was carried out with Image J software. The number of immunopositive cells and integrated optical density (IOD) in the POA and PVN regions of the brain and testes tissues producing positive signals was recorded (four sections per brain and testes from each group). Average IOD values of brain and testes sections from each group were determined as arbitrary unit (AU) thresholds for GnRH-I, ERα, Caspase-3 and p53. Normalized apical fluorescent intensity relative to the control was represented as mean value ± SD per group^44^.

### Biochemical estimations

Four mice from each group were sacrificed and brain, pancreas, and testes were harvested and instantly frozen at − 80 °C for estimation of oxidative stress and antioxidant enzyme activity. Thereafter, the brain, pancreas, and testes were homogenized in 0.02 M Tris–Cl (pH 7.4) and centrifuged at 20,000×*g* for 30 min at 4 °C. The supernatant was collected and kept at − 80 °C for further analysis. Total protein concentration was estimated in the supernatant by Lowry’s method using Bovine serum albumin (BSA) as standard^45^. The whole procedure was performed as previously described by Baghel and Srivastava, 2020^16^.

#### Estimation of H_2_O_2_ concentration

To estimate H_2_O_2_ concentration, 1 ml of 0.01 M phosphate buffer (pH 7.0) was taken in and 50 μl supernatant of the tissues (brain, pancreas or testes) was added. Then glacial acetic acid (1500 μl), and 5% potassium dichromate solution (500 μl), (3:1 v/v) were added and the samples were incubated in a boiling water bath for 5–10 min. Immediately, the reaction mixture was cooled, vortexed and absorbance was measured at 570 nm. H_2_O_2_ concentration was expressed in μM/mg protein^46^.

#### Estimation of malondialdehyde (MDA) levels

For calculating the MDA level in the tissues, 1 ml of 0.5 M Tris-Maleate buffer (pH 5.5) was taken in a test tube and 50 μl of tissue (brain, pancreas or testes) supernatant was added to it. After this, 0.8% of 1.5 ml thiobarbituric acid (TBA) was added and the mixture was kept in a boiling water bath for 10 min. The reaction mixture was cooled at RT and then mixed with n-butanol and pyridine reagent (3:1 v/v). Following this, 1 ml of 1N NaOH was used to stop the reaction, and the absorbance was recorded at 548 nm. An extinction coefficient of 0.152 was used for the calculation of MDA as nmol /mg protein^47^.

#### Measurement of superoxide dismutase (SOD) activity

For measuring SOD activity in the tissues, a reagent comprising 27 ml of 100 mM of PBS (pH 7.8), 1 ml of 2.25 mM nitro blue tetrazolium (NBT), 1 ml of 1 M sodium carbonate, 1.5 ml each of 200 mM L-methionine and 3 mM EDTA was used. Homogenate (50 µl) of the tissue was mixed with 1.35 ml reagent and 200 µl of riboflavin (60 µM) was added for the initiation of the reaction. The reaction mixture was provided exposure to a 20-Watt fluorescent lamp for 10 min and the absorbance was recorded at 560 nm. An identical reaction mixture, kept in the dark, served as blank. The SOD activity was expressed in U/mg protein^48^.

#### Catalase (CAT) activity estimation

For estimating catalase activity in the tissues, the reaction mixture was primed with 1 µmol H_2_O_2_ in 0.05 M sodium phosphate buffer (pH 7.0) and then 50 µl of the sample was added. The reaction was conducted at 25 °C and the absorbance was recorded at 240 nm. The catalase activity was calculated using an extinction coefficient of 0.0436 mM^−1^ cm^−1^ and was expressed as U/mg protein^46^.

#### Glutathione peroxidase (GPx) assay

For estimating the GPx activity in the tissues, 50 µl of supernatant was added to the reaction mixture containing 10 mM sodium azide, 30 mM potassium phosphate buffer, 4 mM reduced glutathione, 2.5 mM H_2_O_2_ and 6 ml distilled water. It was incubated for 5 min at 37 °C and 0.5 ml of 10% tri-chloroacetic acid (w/v) was added for protein precipitation. The reaction mixture was then centrifuged at 700×*g* for 5 min and 0.3 M of 1 ml dipotassium hydrogen phosphate was added to 1 ml of resulting supernatant. To this mixture, 1 ml of Elman’s reagent was added and kept for 3 min at RT. Finally, GPx activity was recorded at 412 nm and expressed as U/mg protein^49^.

#### Glutathione reductase (GR) assay

A reaction mixture comprising 0.2 M phosphate buffer (pH 7.4), 1 M oxidized glutathione, 0.2 M EDTA, and 0.2 M NADPH was prepared. Then, 20 µl homogenate of each tissue was added to the reagent. The oxidation of NADPH was measured as a three-minute decline in the absorbance at 340 nm, and the amount of NADP generated (nmol per minute) at 30 °C was recorded as GR activity and expressed as U/mg protein^50^.

### Enzyme-linked immunosorbent assay (ELISA)

The estimation of testosterone, estradiol and insulin in serum was done by ELISA kits using EIA kit 582701, EIA kit 58225 and EIA kit 589501 respectively, as per the manufacturer’s instructions (Cayman Chemical, USA).

### Statistical analysis

All statistical analyses were performed using a two-way analysis of variance (ANOVA) followed by Bonferroni’s post hoc test. Student’s t-test was applied for comparison between the control and experimental groups individually. The statistical analyses were executed with GraphPad Prism 8.0.2 software (San Diego, CA, USA). A Shapiro–Wilk normality test was used to measure the normality of all datasets. Results represent average values ± SD and were considered statistically significant at *p* < 0.05.

### Ethical approval

All applicable institutional and/or national guidelines for the care and use of animals were followed.

## Results

### Impact of WA on body and testes weight, testicular volume, testis density, and GSI of DM mice

**Figure 1:**
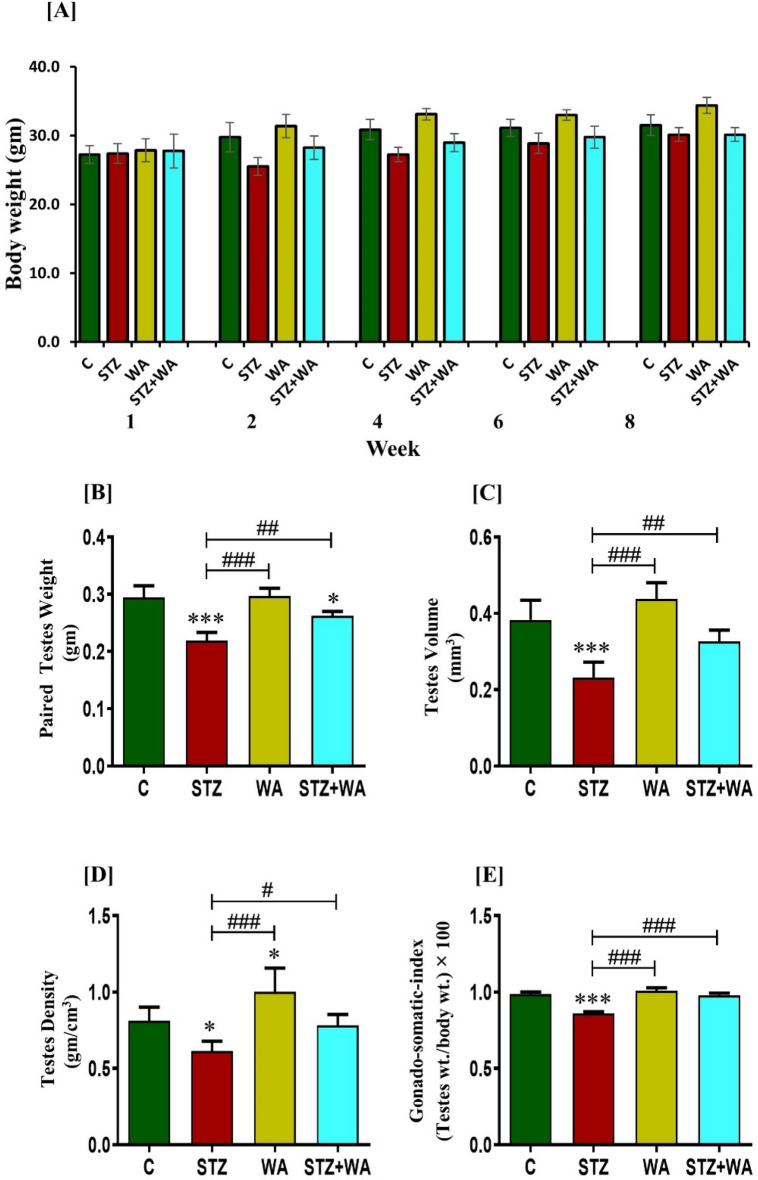

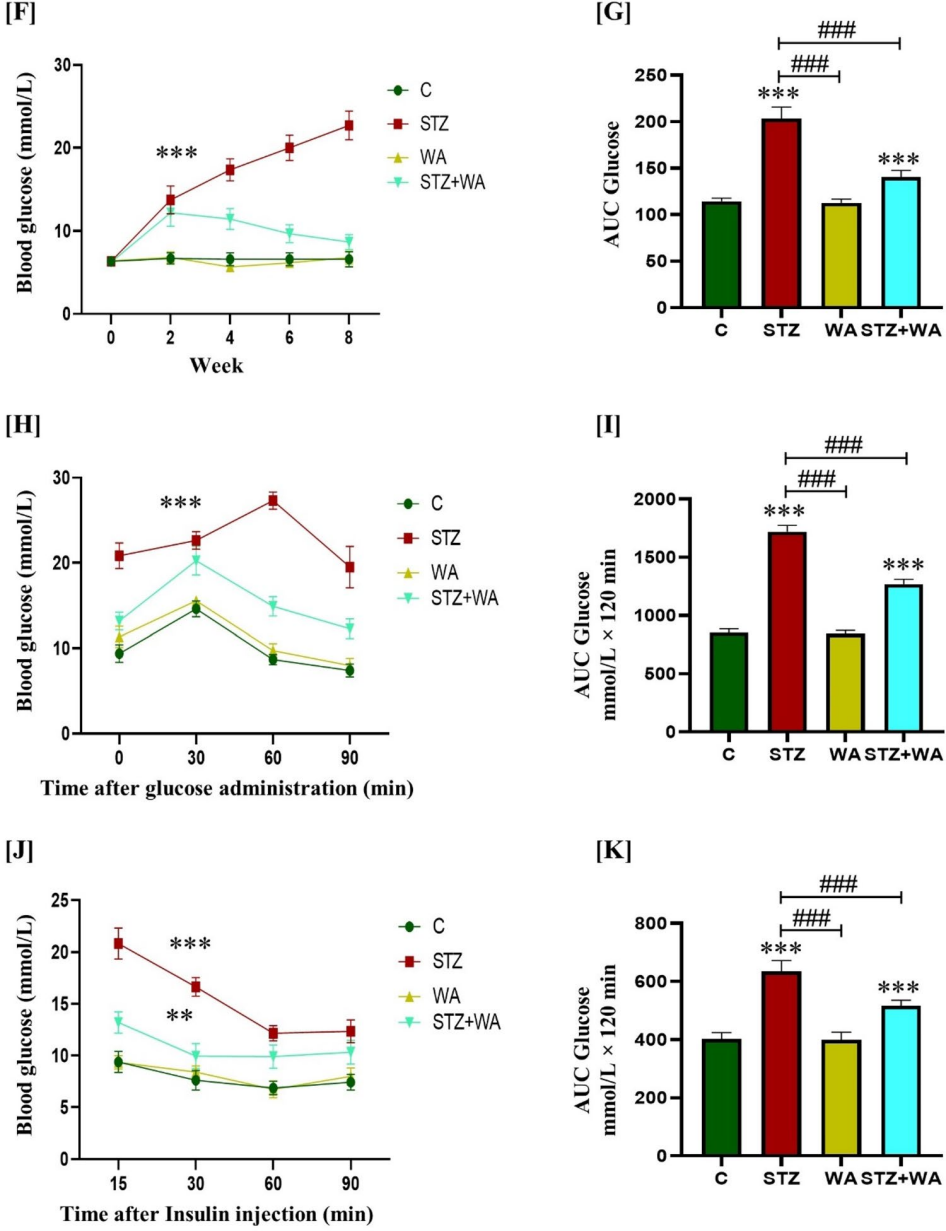
Effect of WA on DM induced reproductive dysfunction; (**A**) Body weight. (**B**) Paired testes weight. (**C**) Testicular volume. (**D**) Testis density. (**E**) Gonado-somatic index. (**F**) Graph showing fasting blood glucose levels weekly and **(G)** Area under curve (AUC), (calculated as a percentage with respect to normal control mice) over 8 weeks. Blood glucose was measured on days 0, 7, 14, 21, 28, 35, 42, 49, 56 and 63. **(H)** Oral glucose tolerance test including. **(I)** AUC after an encounter dose of D-glucose (2 g/kg) for the estimation of glucose tolerance in mice. **(J)** Intraperitoneal insulin (0.75 U/kg) tolerance test including its. **(K)** AUC after an encounter dose of insulin (0.75 U/kg) for the estimation of glucose clearance in mice. Values are presented as mean ± SD, (n = 6), significance of difference is considered as *p* ≤ 0.05 (*p ≤ 0.05, ***p ≤ 0.001) with respect to indicated groups. (*, **, and *** indicated the comparison between C and WA, STZ and STZ + WA; ^#^, ^##^, and ^###^ indicate significant difference from the diabetic (STZ) group at *p* < 0.05, *p* < 0.01 and < 0.001, respectively. [C, Control; STZ, Streptozotocin; WA, Withaferin-A; STZ + WA, Streptozotocin + Withaferin-A].

Over the entire course of the investigation, body weight of animals was recorded weekly. The body weight of STZ-induced diabetic mice decreased slightly as compared to the control group. However, the decrease in body weight was non-significant (*p* > 0.05). After treatment with WA alone, the body weight of the mice was slightly increased as compared to the control, while no increase in the body weight of STZ + WA group was observed (Fig. 1A). Also, there was a slight increase in the testes weight in WA group (0.30 ± 0.014 g) as compared to the control group (0.29 ± 0.021 g). On the other hand, STZ treatment significantly decreased the testes weight (0.22 ± 0.014 g) as compared to the control group, whereas treatment with WA in diabetic conditions significantly (*p* < 0.01) restored the testes weight in STZ + WA group (0.26 ± 0.008 g). Next, we also observed the testicular volume, density and GSI. Paired testicular volume was highest in WA group (0.43 ± 0.042), whereas it was significantly (*p* < 0.01) decreased in STZ induced diabetic group (0.23 ± 0.041) due to STZ-induced diabetes as compared to control (0.38 ± 0.051). WA treatment in STZ + WA during diabetes evidently restored the testicular volume (0.33 ± 0.023) compared to the STZ group. Testicular density was highest in WA group (1.00 ± 0.15), whereas it was significantly (*p* < 0.05) decreased in the STZ group (0.62 ± 0.06) due to the prevailing diabetic conditions compared to control mice (0.81 ± 0.09). WA treatment during diabetes clearly restored the testicular density in STZ + WA group (0.78 ± 0.07), compared to STZ group. Data obtained through GSI was higher in WA group (1.00 ± 0.019), whereas it was significantly (*p* < 0.05) decreased in STZ group (0.86 ± 0.001) in comparison to control mice (0.98 ± 0.014). Thus, WA treatment during diabetes restored the GSI in STZ + WA group (0.98 ± 0.013) (Fig. 1B–E).

### The intercession of WA in DM enhances glucose clearance

Prolonged hyperglycemia during DM results in impaired glucose clearance and is a vital symptom of STZ-induced diabetes leading to insulitis. The glucose levels on weekly basis, OGTT, IPTT as well as their respective AUC values are presented. STZ successfully induced DM was clearly indicated by the progressive rise in the blood glucose levels of the diabetic mice. Enhanced blood glucose levels were noticed in diabetic mice over 1st to 8th week in comparison to the control group. Intraperitoneal treatment of WA in diabetic conditions significantly lowered the blood glucose level in STZ + WA mice as compared to the diabetic group (STZ) (*p* < 0.01). Treatment of WA alone showed no significant change in the blood glucose level. It was noticed that diabetic mice had decreased glucose clearance due to their persistently elevated glucose levels (*p* < 0.001) as compared to the control group (Fig. 1F and G). Remarkably, results from OGTT and IPTT in WA-treated mice further confirmed the anti-diabetic properties of WA as indicated by normal glucose clearance in the treated mice in diabetic condition along with moderate AUC values in contrast to the diabetic group (*p* < 0.001). Furthermore, there was no alteration in the physiological glucose homeostasis in the group treated with WA alone, demonstrating its safety profile (Fig. 1H–K).

### Histological analysis of the pancreas, brain, and testes

#### Impact of WA on pancreatic pathophysiology and STZ-induced DM

**Figure 2:**
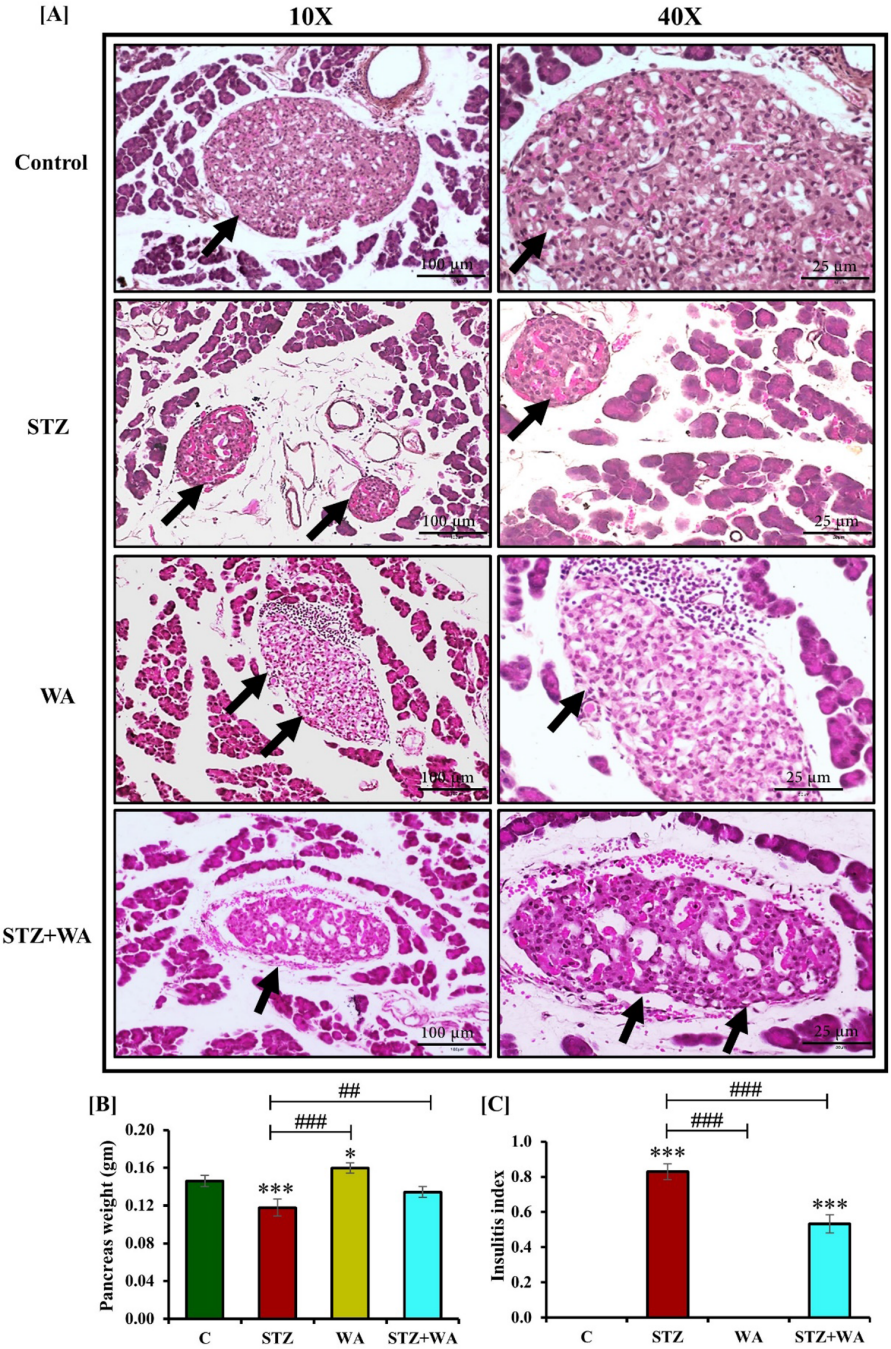
Effect of STZ-induced DM and WA intervention (8 mg/kg/day, i.p.) on histopathophysiology of pancreas and pancreatic insulitis index; (**A)** Illustrative photomicrographs at lower (×10) and higher magnifications (40X) of the pancreatic histology assessed by the H & E staining in Control and all experimental groups viz., STZ; WA and STZ + WA group. Arrows indicate islets of Langerhans and pathological sites of insulitis. **(B)** Graph showing pancreas weight. **(C)** Insulitis index. All values are presented as mean ± SD, (n = 6). The significance of difference is considered as *p* ≤ 0.05 (*p ≤ 0.05, **p ≤ 0.01, ***p ≤ 0.001) with respect to indicated groups. (*, **, and *** indicated the comparison between C and WA, STZ and STZ + WA; ^#^, ^##^, and ^###^ indicate significant difference from the diabetic (STZ) group at* p* < 0.05, *p* < 0.01 and *p* < 0.001, respectively. [C, Control; STZ, Streptozotocin; WA, Withaferin-A; STZ + WA, Streptozotocin + Withaferin-A].

The pancreas weight (0.15 ± 0.06 g) and islets of Langerhans in the control mice were normal. Histopathological observations suggested compact β-cells in pancreatic tissues and there were no signs of insulitis whereas, significant reduction in the pancreas weight along with intense β-cell demolition and insulitis in STZ-induced diabetic mice (0.11 ± 0.009 g) was noticed. Interestingly, WA treatment significantly reduced the β-cell destruction in the pancreas of STZ + WA group mice, with moderate insulitis in contrast to the control group. As compared to the diabetic group, pancreas weight was higher with reduced signs of insulitis in the STZ + WA group (0.13 ± 0.006 g). Inhibition of insulitis in STZ + WA group mice was significant and the WA treatment protected islets of Langerhans showing intense β-cells with normal pancreatic weight (0.16 ± 0.005 g) as compared to the control group (Fig. 2A–C).

#### Impact of WA on brain histology in STZ-induced DM

**Figure 3:**
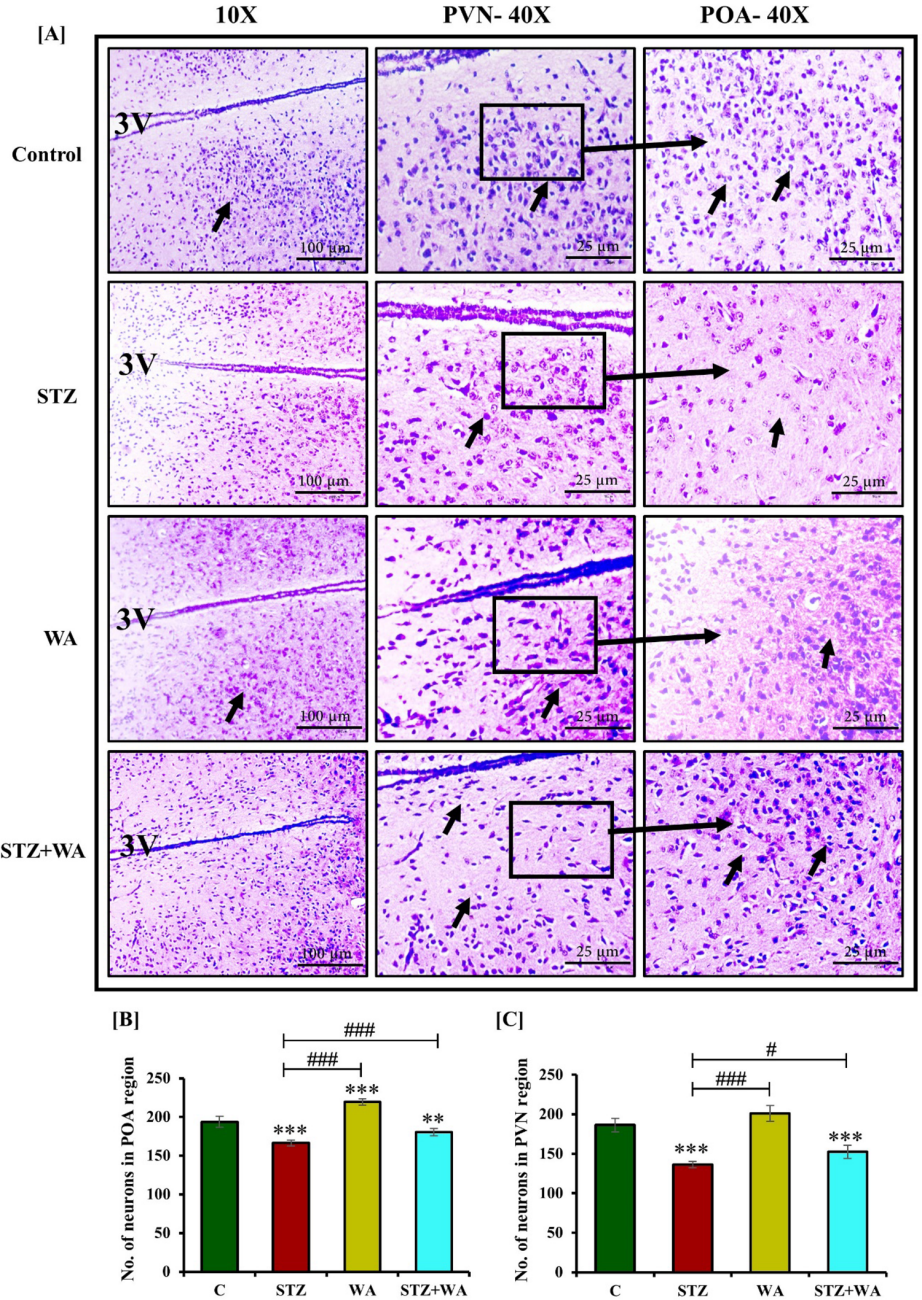
Photomicrographs demonstrating cresyl violet staining in brain; Illustrative photomicrograph at both 10X and 40X magnifications **(A)** STZ mice shows decreased no. of neurons both in POA and PVN region whereas, WA treatment exhibits a significant increase in neuronal number in the POA and PVN region of the brain of STZ + WA group mice. WA administration indicates abundant neurons in both POA and PVN regions. Arrows show neurons in the POA and PVN regions of brain. **(B)** POA and **(C)** PVN regions of brain. Values are presented as mean ± SD, (n = 6). The significance of difference is considered as *p* ≤ 0.05 (*p ≤ 0.05, **p ≤ 0.01, ***p ≤ 0.001) with respect to indicated groups. (*, **, and *** indicated the comparison between C and WA, STZ and STZ + WA; ^#^, ^##^, and ^###^ indicate significant difference from the diabetic (STZ) group at *p* < 0.05, *p* < 0.01 and *p* < 0.001, respectively. [C, Control; STZ, Streptozotocin; WA, Withaferin-A; STZ + WA, Streptozotocin + Withaferin-A].

Large number of neurons in both POA (194 ± 7.1) and PVN (hypothalamic) regions of control mice were observed with Cresyl violet (CV) staining. However, after the induction with STZ, population of the neurons was significantly reduced (*p* < 0.05) in the POA (166 ± 4.0) of diabetic mice. It was elevated post-treatment with WA in the STZ + WA group (180 ± 4.8) as compared to STZ-induced diabetic mice, however, it was lesser than the control group. The neuron population was predominantly visible in the group treated with WA alone (220 ± 3.9) (Fig. 3A–C).

#### Impact of WA on the testis pathophysiology in STZ-induced DM

**Figure 4:**
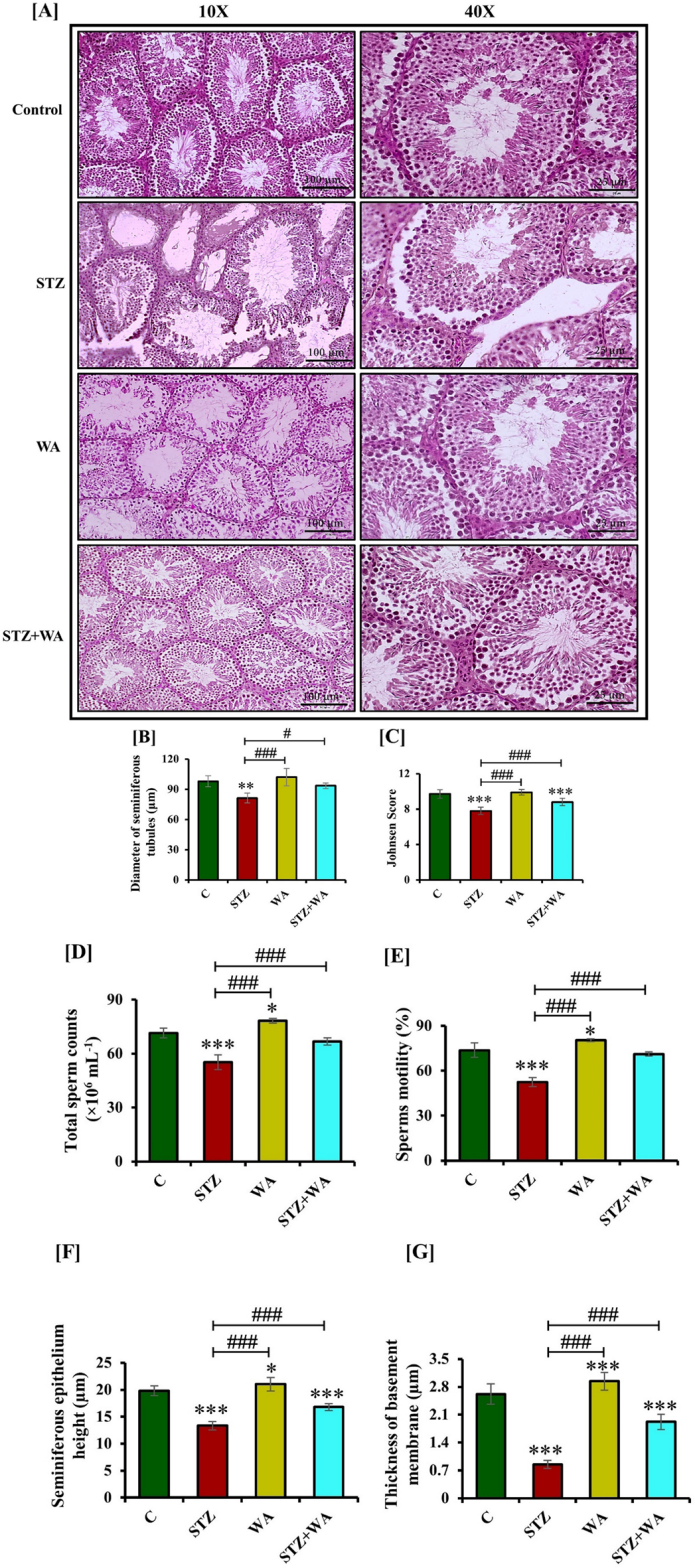

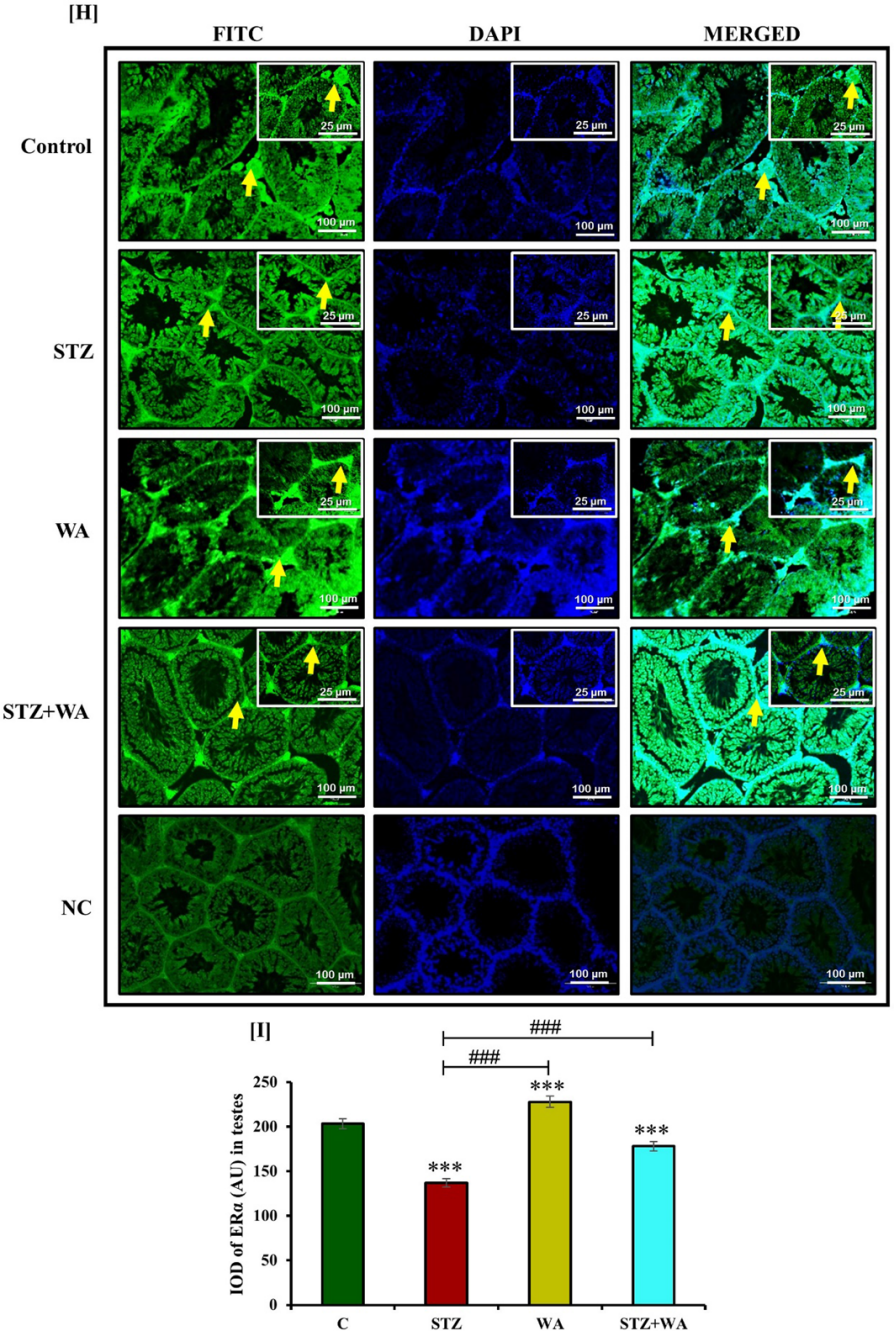
Effect of STZ-induced diabetes on testes histopathology by H & E staining; **(A)** Testis sections of control and WA groups illustrate normal appearance of rounded seminiferous tubules with all testicular cells. In the STZ group, diameter of seminiferous tubules shows significant reductions and the tubules appear distorted. In STZ + WA group, WA treatment shows the restoration among testicular cells in the lumen shown at both 10X and 40X magnifications. Graphs demonstrate. **(B)** Diameters of seminiferous tubules. **(C)** Johnsen score. **(D)** Total sperm count. **(E)** Sperm motility. **(F)** Seminiferous epithelium height. **(G)** Thickness of basement membrane. **(H)** Immuno-fluorescent localization of ERα in testes. Right upper corner shows expression of ERα in testicular cells at higher magnification. **(I)** Graph represents the mean from immune-positive cells by integrated optical density (IOD) in arbitrary units (AU). Values are presented as mean ± SD, (n = 6), significance of difference is considered as *p* ≤ 0.05 (*p ≤ 0.05, **p ≤ 0.01, ***p ≤ 0.001) with respect to indicated groups. (*, **, and *** indicated the comparison between C and WA, STZ and STZ + WA; ^#^, ^##^, and ^###^ indicate significant difference from the diabetic (STZ) group at *p* < 0.05, *p* < 0.01 and *p* < 0.001, respectively. [C, Control; STZ, Streptozotocin; WA, Withaferin-A; STZ + WA, Streptozotocin + Withaferin-A].

Histological observations of the testis showed mature testicular cells with 3–4 layers of spermatogonia with high Johnsen score demonstrating the active reproductive stage in the control group. Sperms were seen in clusters along with head and tail. Also, sperm population and motility were high in the control and WA-treated groups and Leydig cells with round nuclei were seen in the cytoplasm. In the diabetic mice, the diameter of the seminiferous tubules was significantly reduced (81 ± 4.72 µm) as compared to the control (98 ± 5.51 µm) and distortion was noticed between testicular cells. Johnsen score, sperm population and sperm motility were decreased in the diabetic group. Administration of WA increased the diameter of seminiferous tubules (102 ± 8.6 µm) and the Johnsen score. Treatment of STZ-induced diabetic mice with WA possibly increased the Johnsen score by curing damage in testicular cells as indicated by an elevated diameter of the seminiferous tubules (94 ± 2.74 µm) along with moderate sperm population compared to diabetic mice but not up to control group (Fig. 4A–E). The height of epithelium in central and peripheral seminiferous tubules along with the thickness of basement membrane were significantly reduced in the STZ group in contrast to WA treated and the control. It was noticed that after the WA treatment of diabetic group, height of the epithelium in central and peripheral seminiferous tubules and thickness of basement membrane was increased in comparison to the diabetic mice (Fig. 4F and G).

### Immunofluorescence localization of GnRH-I, ERα, and Caspase-3

#### Immunoreactivity of ERα (*ir*-ERα) in testes

Expression of ERα in spermatogonia, spermatocytes, Sertoli cells and Leydig’s cells was maximum in the WA group. Immunoreactivity of ERα was very low in the testicular cells of diabetic mice showing impaired reproduction. WA treatment in diabetic mice helped regain ERα in testicular cells showing its curative effect (Fig. 4H). Fluorescent intensity was decreased for ERα in STZ-induced diabetic mice as compared to the control, while it was enhanced after WA treatment of the diabetic but remained less than the control group. It is noteworthy, that the group treated with WA alone had much higher fluorescent intensity for ERα in testes indicating its role in the reproductive fecundity (Fig. 4I).

#### Immunoreactivity of GnRH-I and ERα receptors in the brain

**Figure 5:**
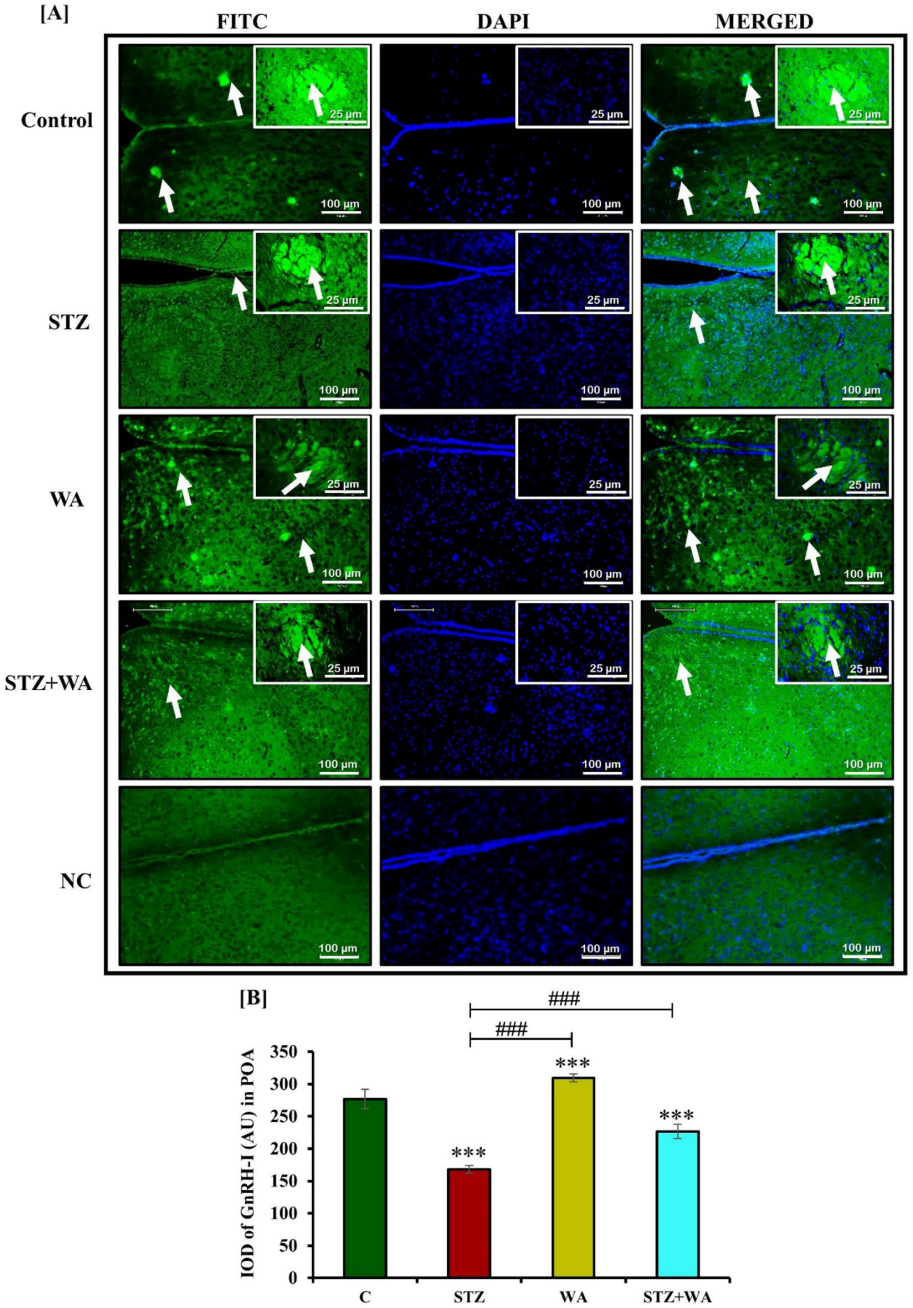

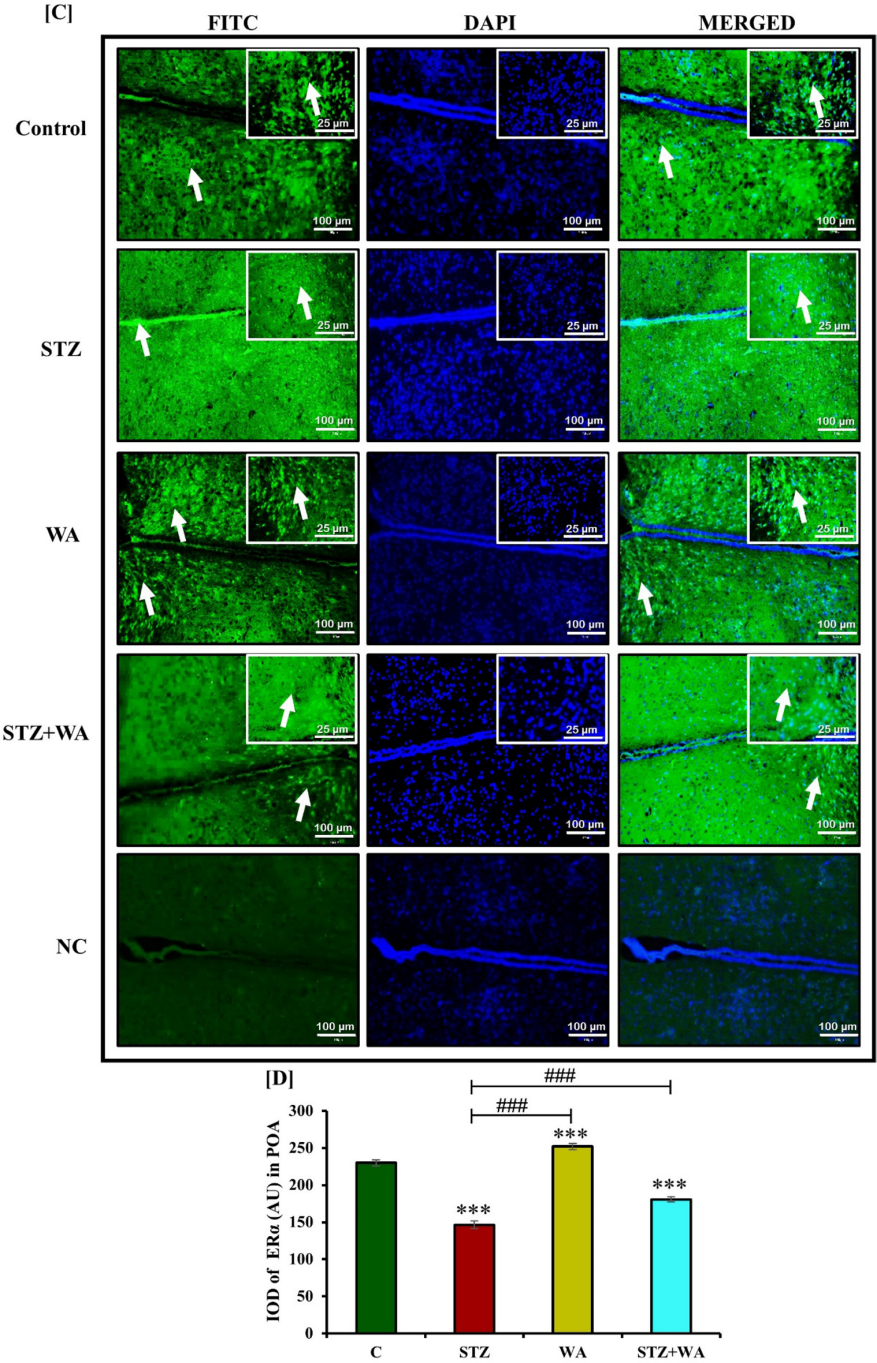
Immuno-fluorescent localization of GnRH-I and ERα receptors in hypothalamic POA and PVN region at both 10X and 40X magnifications; (**A & C)** Diabetic condition reduced *ir*-GnRH-I and *ir*-ERα in both POA and PVN region of brain of mice, whereas C, WA and STZ + WA groups mice showing abundant expression of GnRH-I and ERα. Right upper corner shows expression of GnRH-I and ERα in POA at higher magnification. Arrows indicate the expression of both GnRH-I and ERα in POA and PVN regions. **(B & D)** Graph represents the mean values of integrated optical density (IOD) in arbitrary units (AU) in both POA and PVN regions of brain. Values are presented as mean ± SD, (n = 6), significance of difference is considered as *p* ≤ 0.05 (***p ≤ 0.001) with respect to indicated groups. (*** indicated the comparison between C and WA, STZ and STZ + WA; ^###^ indicates significant difference from the diabetic (STZ) group at p < 0.001. [C, Control; STZ, Streptozotocin; WA, Withaferin-A; STZ + WA, Streptozotocin + Withaferin-A].

We observed the immunoreactivity of GnRH-I and ERα in the neurons of hypothalamic POA and PVN regions. GnRH-I and ERα were well expressed in the hypothalamic POA and PVN regions of the control. Diabetic mice exhibited least immunopositive cells with GnRH-I and ERα in both PVN and POA neurons. On the other hand, administration of WA induced the immunoreactivity of both GnRH-I and ERα receptors as compared to the control. The diabetic group treated with WA (STZ + WA) showed moderate immunoreactivity for GnRH-I and ERα in both the POA and PVN regions of the brain (Fig. 5A and C). In negative control group, immunoreactivity for both GnRH-I and ERα receptors was absent. Semi-quantitative analysis of GnRH-I and ERα receptors was done by measuring fluorescent intensity. Very low fluorescent intensity was observed in POA and PVN regions of diabetic mice for both GnRH-I and ERα receptors whereas, it was significantly increased after the treatment with WA in diabetic group in both POA and PVN regions. Thus, it was concluded that WA treatment enhanced fluorescent intensity in both POA and PVN regions (Fig. 5B and D).

#### Immunoreactivity of Caspase-3 and p53 in testes

**Figure 6:**
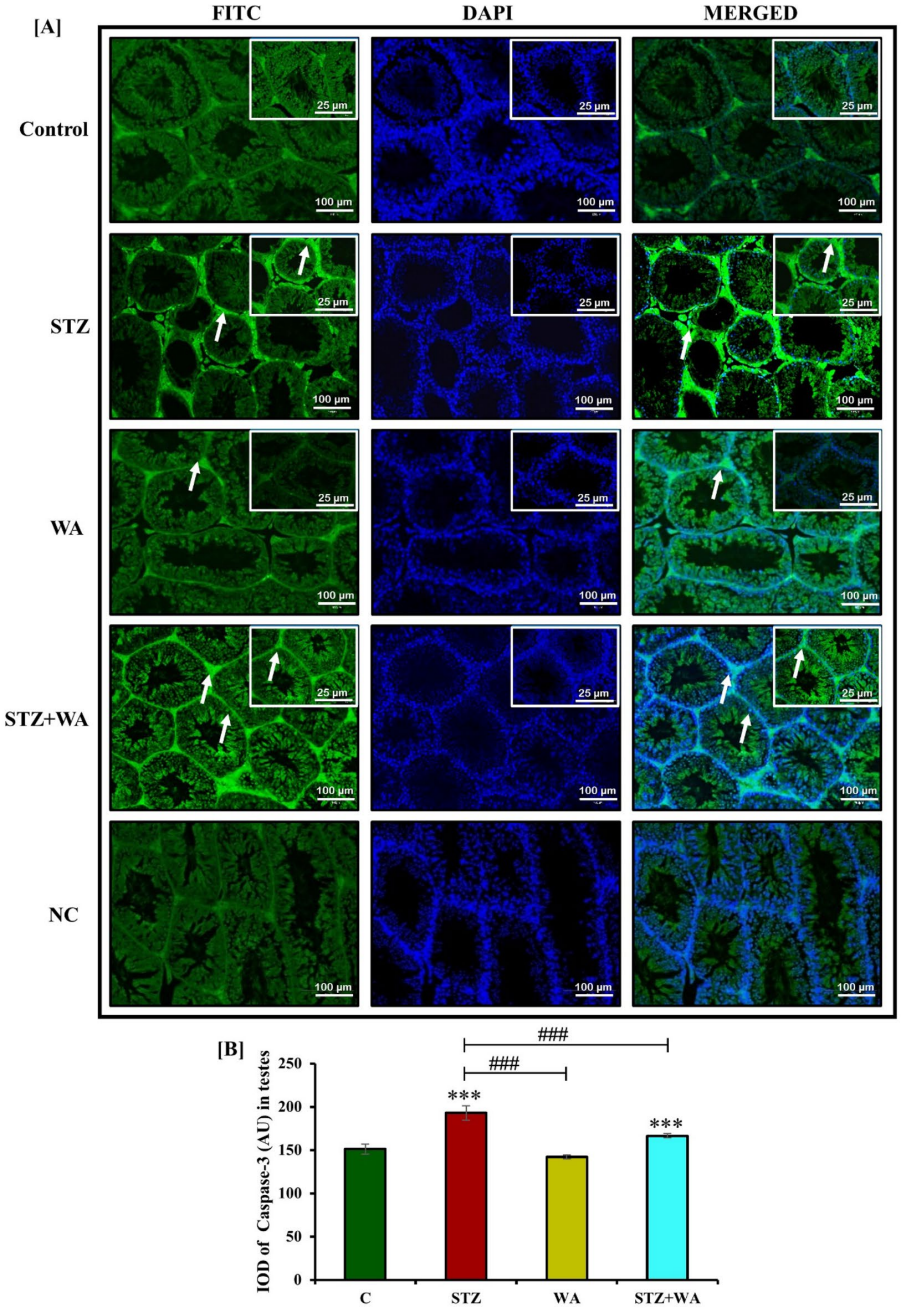

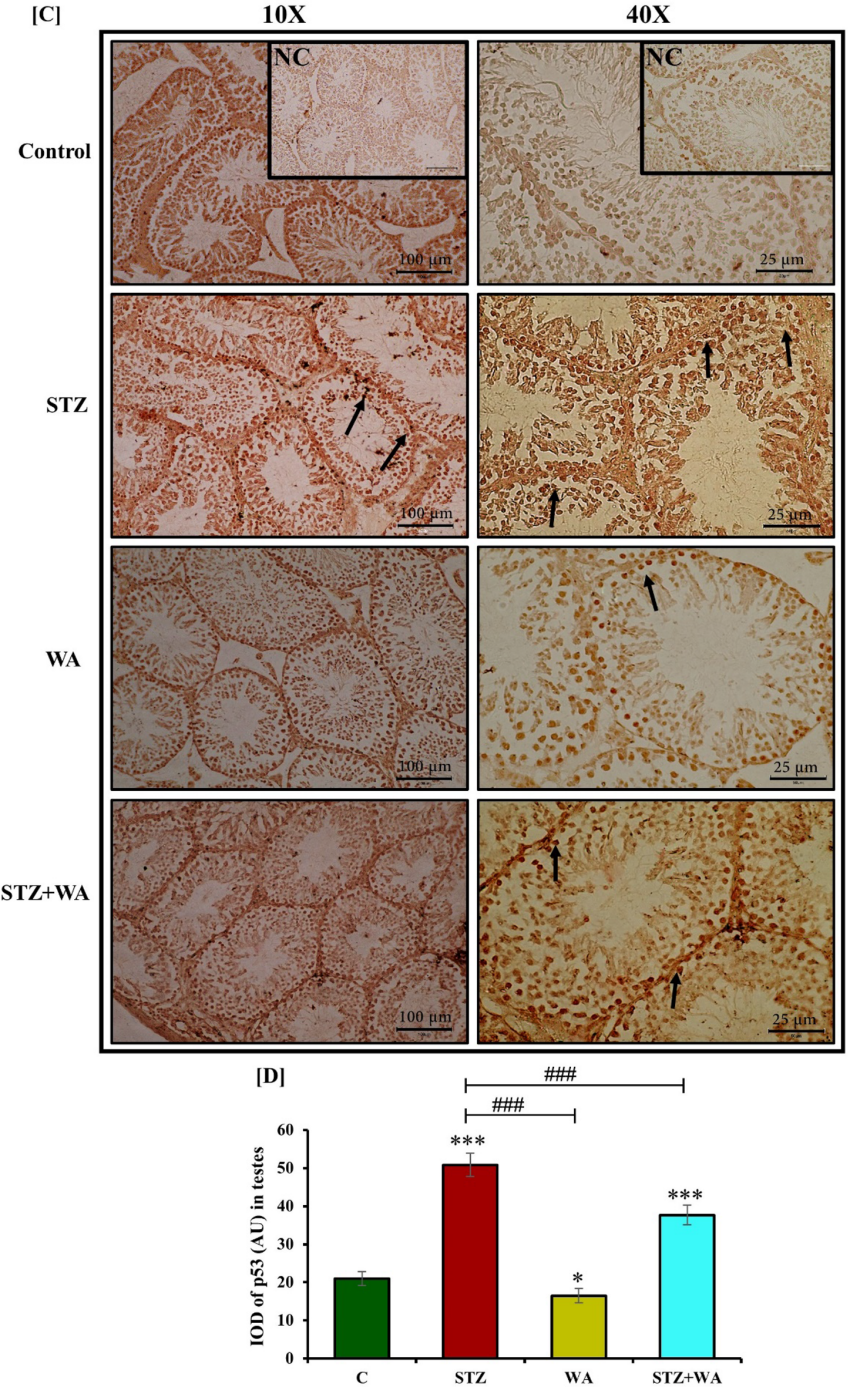
Immuno-fluorescence localization of Caspase-3 and p53 in testicular cells of mice; (**A & C)** STZ-induced diabetic mice testis illustrates highest *ir*-Caspase-3 and *ir*-p53 in spermatogonial cells, whereas WA treatment testis shows weak immunoreactivity in all stages of testes in STZ + WA group at both 10X and 40X magnifications. Control and WA-treated mice testes have low expression for Caspase-3 and p53 in testicular cells. Arrows indicate the immunoreactivity of both Caspase-3 and p53 in testicular cells. **(B & D)** Graph represents the mean from immune-positive cells by integrated optical density (IOD) in arbitrary units (AU). Values are presented as mean ± SD, (n = 6), significance of difference is considered as *p* ≤ 0.05 (*p ≤ 0.05, ***p ≤ 0.001) with respect to indicated groups. (* and *** indicated the comparison between C and WA, STZ and STZ + WA; ^###^ indicate significant difference from the diabetic (STZ) group at *p* < 0.05, *p* < 0.01 and *p* < 0.001, respectively. [C, Control; STZ, Streptozotocin; WA, Withaferin-A; STZ + WA, Streptozotocin + Withaferin-A].

Caspase-3 and p53 are considered apoptotic markers in the testicular cells. The control and WA group had the lowest *ir*-Caspase-3 and *ir*-p53 in testicular cells. Immunoreactivity of Caspase-3 and p53 revealed a similar pattern indicating apoptosis in the testicular cells of diabetic mice demonstrating highest apoptosis. Caspase-3 and p53 had moderate expression in the testicular cells of STZ + WA group mice. WA treatment could overcome the testicular apoptosis in the diabetic mice showing a reduction in Caspase-3 and p53 in STZ + WA group (Fig. 6A and C). Fluorescent intensity for Caspase-3 and p53 was highest in testicular cells of diabetic mice demonstrating testicular apoptosis compared to the control group. Thus, WA treatment could overcome the testicular apoptosis with reduction in Caspase-3 and p53 in STZ + WA group mice (Fig. 6B and D). In the group treated with WA alone, minimum fluorescent intensity for both Caspase-3 and p53 indicated the negligent presence of these apoptotic markers.

### Estimation of oxidative stress and anti-oxidant enzyme activity in the brain, pancreas, and testes of DM mice

#### Lipid peroxidation

**Figure 7:**
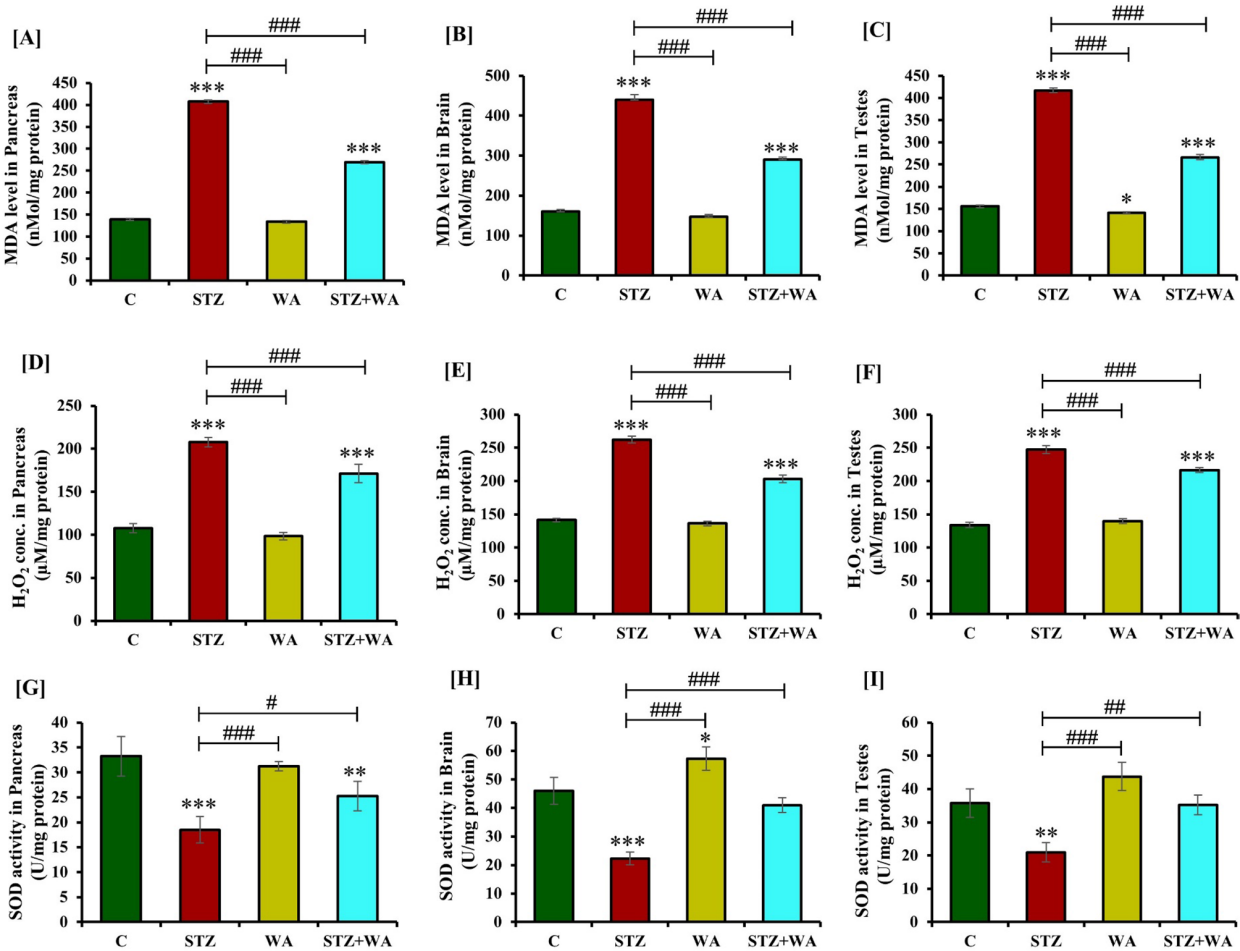

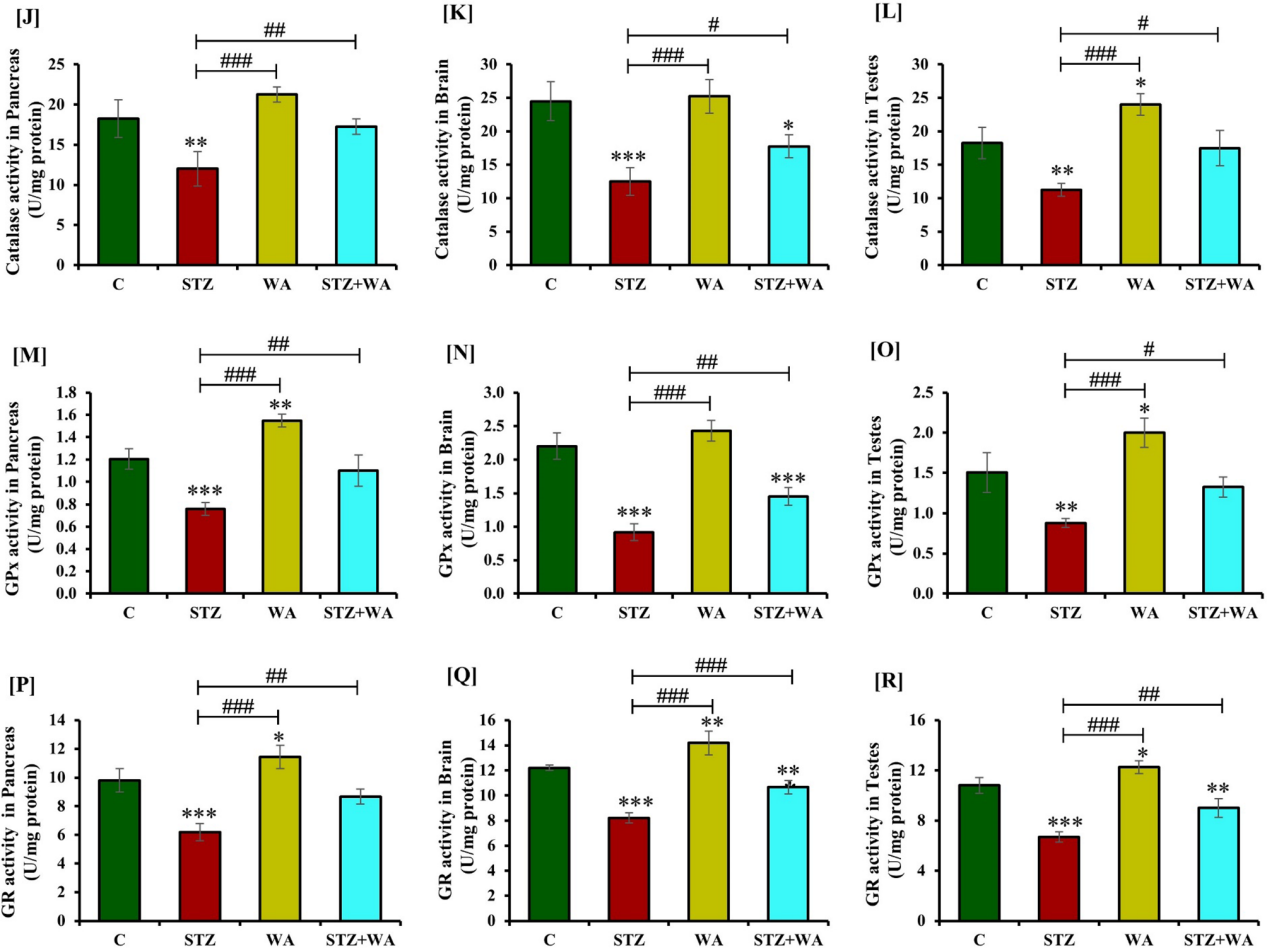
Effect of WA on STZ-induced DM on pancreas, brain and testicular oxidative stress parameters; (**A–C)** MDA levels, **(D–F)** H_2_O_2_ concentration, **(G–I)** SOD activity, **(J–L)** CAT activities, **(M–O)** GPx activity, and **(P–R)** GR activity respectively. Values are presented as mean ± SD, (n = 4), significance of difference is considered as *p* ≤ 0.05 (*p ≤ 0.05, **p ≤ 0.01, ***p ≤ 0.001) with respect to indicated groups. (*, **, and *** indicated the comparison between C and WA, STZ and STZ + WA; ^#^, ^##^, and ^###^ indicate significant difference from the diabetic (STZ) group at *p* < 0.05, *p* < 0.01 and *p* < 0.001, respectively. [C, Control; STZ, Streptozotocin; WA, Withaferin-A; STZ + WA, Streptozotocin + Withaferin-A].

Diabetic mice (STZ group) had higher MDA levels in the pancreas, brain, and testes in contrast to the control mice. Group treated with WA alone showed a non-significant reduction in MDA levels in these tissues. The STZ + WA group mice exhibited significantly (*p* < 0.05) low MDA levels in the brain, pancreas, and testes as compared to the STZ group indicating the ameliorative effect of WA (Fig. 7A–C).

#### Hydrogen peroxide level

The H_2_O_2_ level was significantly (*p* < 0.001) increased in the pancreas, brain, and testes of the diabetic (STZ) group indicating high oxidative stress. In the diabetic group treated with WA (STZ + WA), the amount of H_2_O_2_ in the pancreas, brain and testes was low as compared to the STZ group (Fig. 7D–F).

#### Superoxide dismutase activity

STZ significantly (*p* < 0.001) reduced the activity of SOD in the pancreas, brain, and testes of the diabetic (STZ) mice whereas, the control group had high SOD activity in these organs. WA treatment significantly (*p* < 0.001) increased the SOD activity in these organs in diabetic mice as compared to the STZ group, however, it was lower than the untreated control group. Similarly, treatment of control mice with WA also elevated the SOD activity in the pancreas, brain, and testes as compared to the diabetic group (Fig. 7G–I).

#### Catalase activity

We observed significantly (*p* < 0.001) low CAT activity in the pancreas, brain, and testes of the diabetic (STZ) mice, whereas CAT activity was high in these organs of the control group. Administration of WA in healthy mice resulted in a non-significant increase in CAT activity in these organs. Significant increase (*p* < 0.05) in the CAT activity in these organs was noticed compared to STZ group in the diabetic mice given WA (STZ + WA) treatment (Fig. 7J–L).

#### Glutathione peroxidase activity

The GPx activity was significantly decreased (*p* < 0.001) in the pancreas, brain, and testes of the STZ group in comparison to the control group. WA treatment of healthy non-diabetic mice elevated GPx activity in these organs. Similarly, the GPx activity was significantly (*p* < 0.05) enhanced in the pancreas, brain, and testes of the STZ + WA group as compared to the STZ group, indicating the positive effect of WA treatment (Fig. 7M–O).

#### Glutathione reductase activity

As compared to the control group, the GR activity was significantly (*p* < 0.001) declined in the pancreas, brain, and testes of the diabetic (STZ) group. Individual treatment with WA significantly augmented GR activity in the pancreas, brain, and testes in contrast to the control group. Similarly, GR activity was significantly enhanced in these organs of the STZ + WA group as compared to the diabetic (STZ) group (Fig. 7P–R).

#### Hormonal analysis

**Figure 8:**
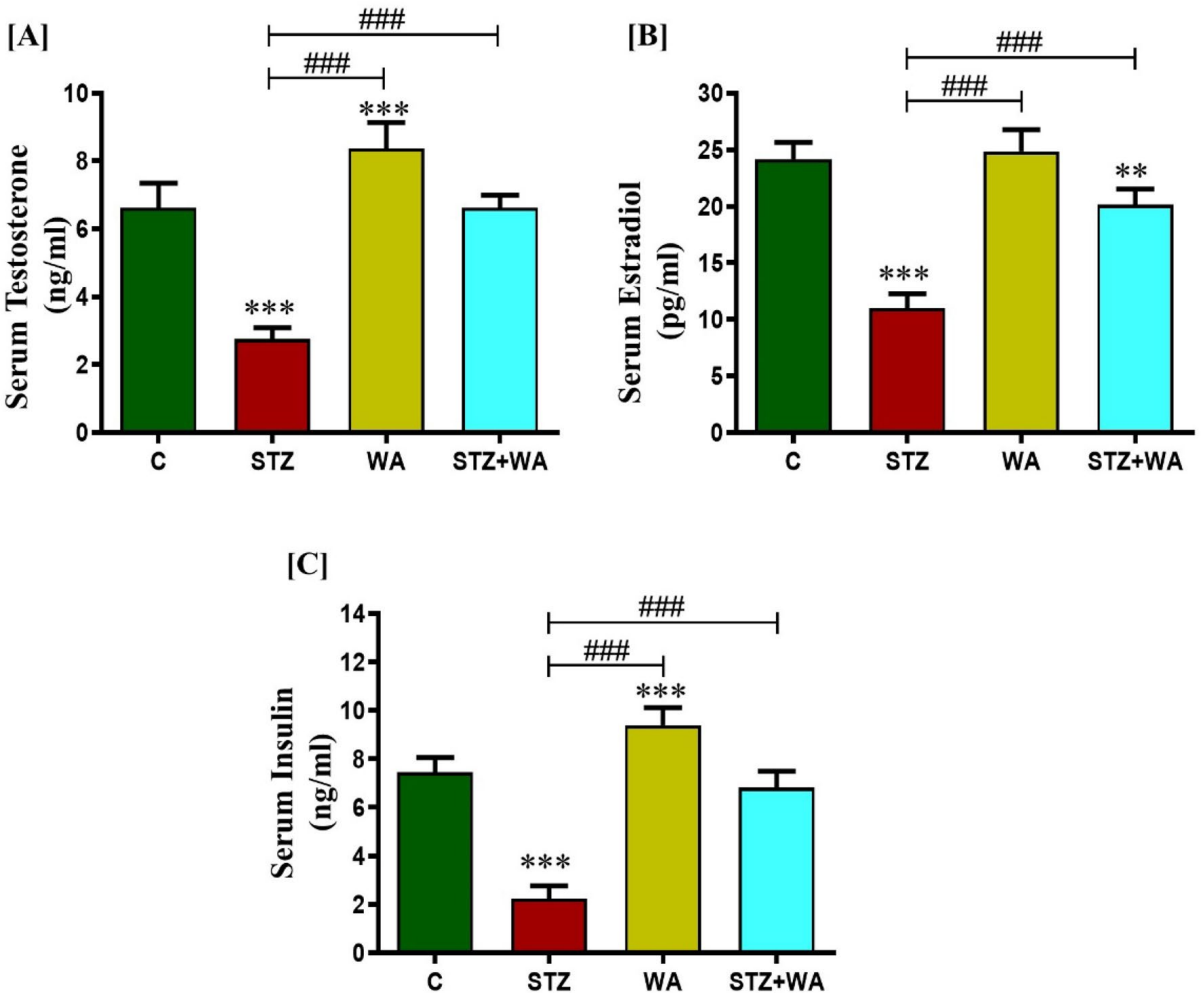
Effects of WA on STZ-induced DM on serum hormone levels; **(A)** Serum Testosterone, **(B)** Estradiol and, **(C)** Insulin levels. All the above hormone levels were significantly lowered in the STZ group as compared to the treatment groups. Values are presented as mean ± SD, (n = 6), significance of difference is considered as *p* ≤ 0.05 (*p ≤ 0.05, **p ≤ 0.01, ***p ≤ 0.001) with respect to indicated groups. (**, and *** indicated the comparison between C and WA, STZ and STZ + WA; ^###^ indicates significant difference from the diabetic (STZ) group at *p* < 0.001. [C, Control; STZ, Streptozotocin; WA, Withaferin-A; STZ + WA, Streptozotocin + Withaferin-A].

Compared to the control group, significant decline in the serum insulin levels (*p* < 0.001) of the diabetic mice (STZ group) was observed, showing effective induction of the DM. In contrast, the WA treatment significantly enhanced insulin levels as compared to diabetic mice. WA significantly elevated the insulin levels in the diabetic mice i.e., STZ + WA group, compared to both control as well as diabetic group. DM reduced both circulating testosterone and estradiol levels in STZ mice in contrast to the control or untreated group, while WA treatment significantly (*p* < 0.001) augmented the levels of both circulating testosterone and estradiol hormones in the STZ + WA group. Administration of WA alone slightly increased the circulating testosterone and estradiol compared to the control group (Fig. 8A–C).

## Discussion

In the present study, we investigated the ameliorative effect of WA on the DM-induced reproductive dysfunctions mediated by ERα in the brain and testicular cells. Prolonged DM-induced reproductive dysfunctions cause impaired spermatogenesis, low functionality and motility of sperms along with low circulating sex steroid levels. Firstly, STZ-induced diabetic condition in mice was confirmed by high fasting glucose levels, OGTT, IPTT, decreased insulin level, and destruction of beta cells of the pancreatic islets of Langerhans along with the presence of insulitis. Similarly, it was also observed that diabetes vastly affected the reproductive performance of mice mediated by a significant decline in sex steroid levels i.e., testosterone and estradiol along with declined antioxidant enzyme activities in the brain, pancreas and testes. Furthermore, low circulating sex steroid levels also lowered the GnRH-I and ERα in the brain and ERα in the testicular cells of STZ-induced diabetic mice. On the other hand, WA markedly lowered the DM-induced reproductive dysfunction mediated by ERα by enhancing the physiological conditions of the mice that include enhanced testes and pancreas weight, increased GSI as well as lowering the level of fasting glucose, glucose intolerance and oxidative stress.

GnRH-I regulates reproductive activity by activating the hypothalamic–pituitary–gonadal (HPG)-axis. GnRH-I, the prime regulator of reproductive activity, also controls the gonadotropin release from the pituitary and consequently, testicular functions. This axis also includes neuroendocrine complexes that integrate variations of external and internal inputs to synchronize reproductive fitness^51^. The accurate functioning of the HPG-axis is pivotal for normal androgenic and reproductive activity. Studies have also revealed that interruption in the HPG-axis is responsible for the induction of DM^52^. The impaired function of the HPG-axis reduces the levels of testosterone and estradiol in diabetic mice. In turn, the low levels of circulatory testosterone and estradiol decrease the immunoreactivity of ERα in POA and PVN region of the brain and also in testicular cells, predominantly in Leydig cells. It is expected that the low levels of sex steroids result in the low weight, volume, density of testis, low GSI, reduced diameter of seminiferous tubule, low Johnsen score, thin basement membrane around the seminiferous tubules, and decreased sperm count leading to hampered spermatogenesis^53^. With regard to the effects of insulin on the HPG-axis, it has already been demonstrated that insulin receptors and the associated signalling pathway mediate these effects. Insulin signalling in the brain and fertility were linked with brain-specific insulin receptors in knockout mice^54^.

Oxidative stress is caused by physiological metabolisms such as the polyol pathway, protein glycosylation and glucose auto-oxidation that produce reactive oxygen species (ROS) in the tissues. It is confirmed that diabetes causes the over-activation of the aforementioned pathways which results in a high degree of ROS generation in the tissues^55^. Additionally, data from our results also showed the highest oxidative stress in the brain, pancreas and testes of the diabetic mice leading to high H_2_O_2_ and MDA levels. Also, it is evident that levels of oxidative stress can damage the DNA of germ cells mediated by overexpression of Caspase-3 and p53 in diabetic conditions resulting in reproductive dysfunction^56^. In the present study, we also found the high immunoreactivity of Caspase-3 and p53 in testicular cells, particularly in germ and Leydig cells of diabetic mice (STZ), as compared to other experimental groups exhibiting testicular apoptosis. Testicular apoptosis occurs predominantly due to elevated secretion of gonadotropin inhibitory hormone (GnIH) from the hypothalamus which induces p53-dependent Bax-Caspase-3 expression in the testicular cells^43^. It is known that enhanced oxidative stress induces apoptosis of germ cells, which in turn, induces testicular damage in diabetic mice^57^. Key antioxidant enzymes such as, SOD, GPx, and CAT substantially eradicate free radicals in diabetic conditions. Furthermore, our study also confirmed that H_2_O_2_ and MDA levels were increased and antioxidant enzymes such as SOD, CAT and GPx levels were reduced in the brain, pancreas and testes of diabetic mice as compared to the control group. Similarly, increased level of ROS in diabetes has a vigorous influence on the sperm population due to the presence of different polyunsaturated fatty acids in the cell membrane of spermatozoa^58^. Several studies have proven that consuming anti-oxidative substances can diminish the oxidative stress in the male reproductive system in diabetic conditions. Subsequently, in diabetic conditions, oxidative imbalance is a primary pathophysiological state in the testis. Oxidative stress negatively affects the reproductive activity of diabetic mice mediated by low expression of ERα in testicular cells along with lesser sperm population, motility and morphology.

The low expression of ERα in the brain and testes might be triggered by the accumulation of Caspase-3 and p53 in testis. In brain, high lipid peroxidation and H_2_O_2_ levels indicated oxidative stress which decreased the activities of antioxidant enzymes like SOD, GPx and CAT, as well as reduced neuronal numbers in POA and PVN regions of the diabetic mice. WA is known to have several important medicinal properties and confers anti-cancer, anti-apoptotic, anti-stress, sedative, anti-inflammatory, and neuroprotective effects. Interestingly, in our study, we also observed that administration of WA in healthy and diabetic (STZ + WA) animals proved very effective in plummeting oxidative stress, glucose levels and elevating endogenous testosterone and estradiol, thereby resulting in the improvement of reproductive health. The thickness of basement membrane plays an important role in spermatogenesis, serving as a proliferation place for spermatogonia and maintenance for the phenotypic differentiation of Sertoli cells. Reproductive health of WA-treated mice was observed with a high Johnsen score, thick basement membrane around the seminiferous membrane and increased sperm population resulting in healthy spermatogenesis. Moreover, this study is in agreement with the previous findings that WS containing a rich source of potent antioxidants, has the potential to reduce oxidative stress by enhancing the antioxidant enzyme activity^59^. WA is used as the first-line treatment for various ailments in the well-acclaimed Indian Ayurvedic medicinal systems. Results from our data also commended that WA, a steroidal lactone and the major bioactive metabolite of WS, modulates endogenous sex steroids as testosterone and estradiol. Further, endogenous estradiol stimulates the level of ERα in brain and testes of WA-treated mice in DM conditions. Finally, WA treatment in diabetic mice helped in overcoming the oxidative stress and simultaneously reduced testicular apoptosis. WA-treated mice exhibited increased serum sex steroids, insulin level, and ERα in brain and testes, indicating the reinstatement of a healthy reproductive state.

## Conclusion

**Figure 9:**
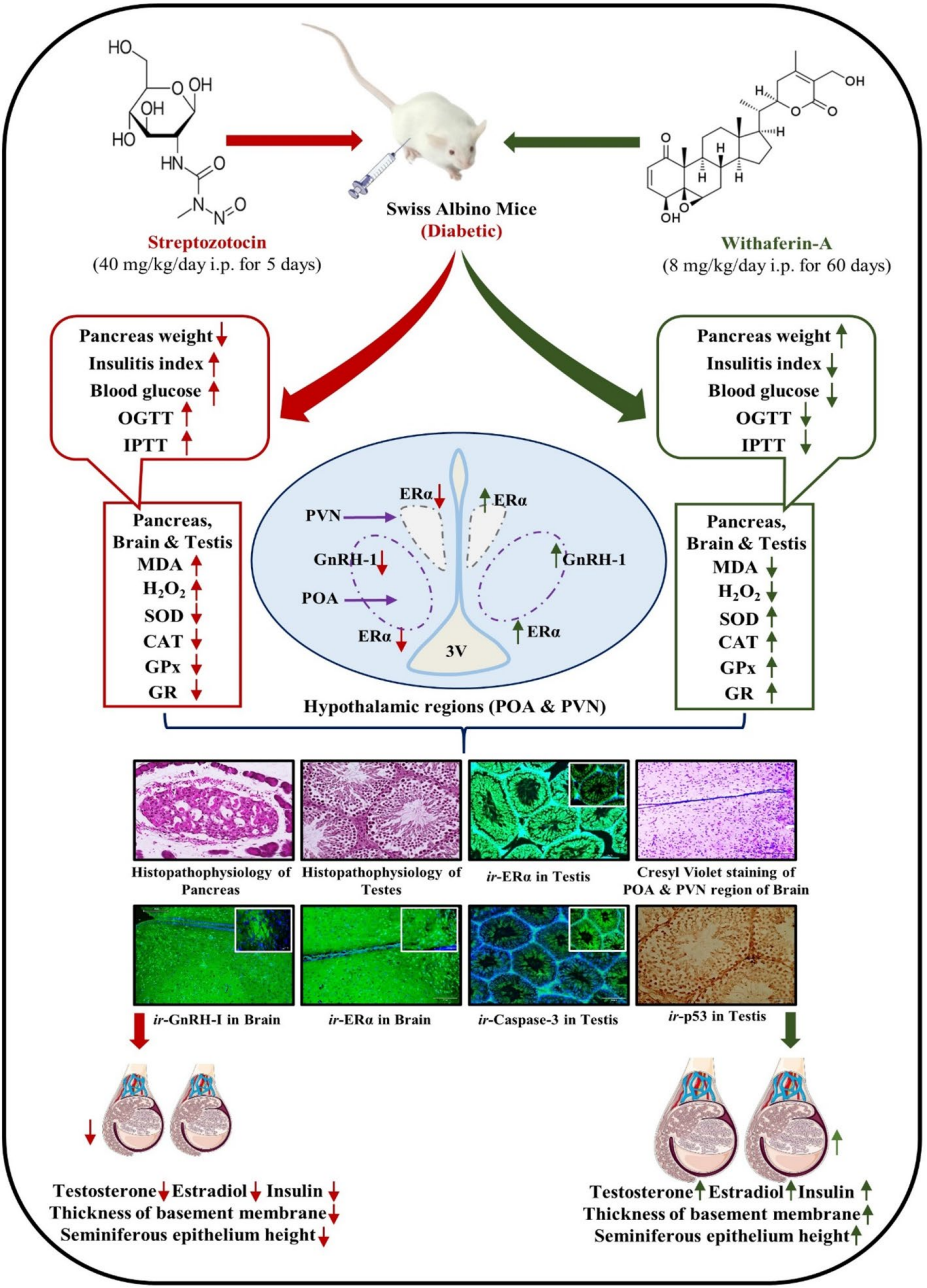
Summary diagram illustrating STZ-induced DM triggered reproductive dysfunction and amelioration by Withaferin-A in Swiss albino mice.

In the current study, synchronized treatment of WA could efficiently ameliorate the diabetes-induced testicular apoptosis mediated by Caspase-3 and p53 along with preventing the diabetogenesis. WA helps in overcoming diabetes-triggered reproductive dysfunction by enhancing the endogenous testosterone and estrogen, as well as increasing immunoreactivity of GnRH-I and ERα receptors in the brain and ERα receptors in the testes. Thus, it can be concluded that WA may be used as an effective strategy in the amelioration of DM-induced reproductive dysfunction. It is an important revelation that WS herbal preparations have the potential to overcome diabetes-induced reproductive disorders. The present study conclusively supports the possible use of WA as a health supplement and shows that it can dramatically lower the reproductive impairment caused by STZ-induced DM (Fig. 9). To the best of our knowledge, we for the first time, elucidated the role of WA in alleviating diabetes-induced reproductive dysfunction in male mice. This study also provides insight into the reproductive alterations occurring in the male reproductive organs due to DM.

## Data Availability

The authors confirm that all data generated during and/or analysed to support the findings of this study are available within the article.

## Abbreviations

AU: Arbitrary unit
AUC: Area under the curve
CAT: Catalase
CV: Cresyl violet
DAPI: 4′, 6-Diamidino-2-phenylindole
DMSO: Dimethyl sulfoxide
ED: Erectile dysfunction
ELISA: Enzyme-linked immunosorbent assay
ERα: Estrogen receptor alpha
ERβ: Estrogen receptor beta
ERs: Estrogen receptors
ESR-KO: Estrogen receptor knock-out
GPx: Glutathione peroxidase
GnRH: Gonadotropin-releasing hormone
GSI: Gonado-somatic-index
GR: Glutathione reductase
H & E: Hematoxylin and Eosin
H_2_O_2_: Hydrogen peroxide
HPG: Hypothalamic–pituitary–gonadal
*ir*: Immunoreactive
IHC: Immunohistochemistry
IOD: Integrated optical density
IPTT: Intraperitoneal insulin tolerance test
MDA: Melonaldehyde
NGS: Normal goat serum
OGTT: Oral glucose tolerance test
PBS: Phosphate buffer saline
POA: Preoptic area
PVN: Paraventricular nucleus
SOD: Superoxide dismutase
STZ: Streptozotocin
DM: Diabetes mellitus
TBA: Thiobarbituric acid
WA: Withaferin A
WS: *Withania somnifera*
